# Improved survival in patients admitted to ICU with multiple myeloma: a retrospective cohort analysis

**DOI:** 10.1016/j.aicoj.2026.100100

**Published:** 2026-06-13

**Authors:** Sabrine Nakaa, Leo Caillot, Akli Chermak, Helene Kemp, Stéphanie Harel, Michael Darmon, Elie Azoulay, Virginie Lemiale

**Affiliations:** a Medical ICU, Saint Louis Hospital, 1 Avenue Claude Vellefaux, 75010 Paris, France; b Immuno-hematology, Saint Louis Hospital, 1 Avenue Claude Vellefaux, 75010 Paris, France

**Keywords:** Multiple myeloma, Outcome, Intensive care, Immunosuppressed host

## Abstract

**Background:**

In the recent years, multiple myeloma (MM) has been associated with long-term survival. Life-threatening complications may occur in those patients leading to high risk of ICU admission. Within the same period, survival of critically ill patients with malignancy improved. The aim of this study was to evaluate severity and outcome in patients with MM admitted to ICU within the last 16 years.

**Methods:**

In this monocentric retrospective study, patients with MM admitted to ICU within two periods (2007−2015) and (2016−2023) around major therapeutic changes, were included. Patients from two periods were compared in terms of short and long terms outcomes. In the recent period, factors associated with mortality were assessed by multivariate analysis.

**Results:**

During the first period (2007−2015), 199 patients were included and compared to 229 patients admitted within the second period (2016−2023). Median delay from MM diagnosis to ICU was 25.9 (2.2−70.6) months and 82 (19.2%) patients were newly diagnosed patients. MM classified as high risk concerned 119 (52%) or Stade III Salmon-Durie for 142 (71.4 %) patients. SOFA score on Day 1 was 5 (2−7). During ICU stay, 115 (26.9%) patients needed invasive mechanical ventilation, 105 (24.5%) patients received vasopressors and 97 (22.7%) had renal replacement therapy. Median length of ICU stay was 3 (2−6) days. Reason for ICU admission was different between the two periods: Shock was more frequent in the second period (29.7% vs 13.7%) whereas acute respiratory failure was more frequent in the first period (35.2% vs 46.7%) (p < 0.001). ICU and one-year mortality rates were respectively 12.1% (n = 50) and 40.6% (n = 170). Mortality rates, adjusted on age, comorbidities, more than 2 lines treatment, time between hospital and ICU admission >1 day, kidney amyloidosis, SOFA score at ICU admission and reason for ICU admission, were lower in the recent period compared to the first period (0.68 (0.49−0.95), p = 0.02).

**Conclusions:**

Survival in MM patients admitted to ICU improved in the recent years. Particularly, patients who were not previously heavily treated had better outcome and should be admitted to ICU.

## Introduction

Multiple myeloma (MM) is plasma cells malignancy, associated with bone lesions, kidney failure, and infectious or metabolic emergencies. Over the past two decades, the prognosis of patients with MM has substantially improved due to the introduction of new agents, including proteasome inhibitors, immunomodulatory drugs, and CD38 monoclonal antibodies such as daratumumab, and more recently T cell engagers including bispecific antibodies and CAR-T cells, and risk-adapted treatment strategies [1]. Even for patient with relapsed or refractory MM, these advances lead to an extended survival, with high level of quality of life [[2], [3], [4], [5], [6]].

As a result, a high proportion of those patients may experience acute life-threatening complications leading to intensive care unit (ICU) admission [[7], [8], [9]]. These complications may arise from the MM itself—such as hypercalcemia, kidney failure, and sepsis—or from treatment-related toxicity, including cytopenia, infections, and cardiopulmonary complications [1,7,10]. Historically, critically ill MM patients had high mortality rates, and ICU admission was often withheld in advanced cases [11]. However, evolving ICU practices and hematology-ICU collaborations have led to broader admission criteria and potentially better outcomes. A previous study published in 2009 demonstrated increased survival between three periods from 1990 to 2006 [11]. However, those periods did not include the recent major change in treatment.

Moreover, recent studies demonstrated that early ICU admission, oxygenation strategies, and chemotherapy during ICU has been associated with improved survival in cancer patients [12,13]. Whether such gains extend to critically ill MM patients in the modern therapeutic era remained unclear. Moreover, while prior studies have emphasized hospital mortality, longer-term outcomes such as one-year survival may better reflect the trajectory of these complex patients.

The objective of this study was to assess changes in ICU admission patterns, management strategies, and outcomes in MM patients over a 17-years period, with a focus on the impact of modern treatments introduced after 2015. We also sought to identify clinical factors associated with mortality in this population.

## Patients and methods

### Study design and setting

We conducted a retrospective monocentric cohort study in a medical ICU in Paris. All patients with MM admitted to ICU between January 2007 and December 2023 were included in the cohort. The ICU was a 12-beds unit until 2020 and then became a 20-beds unit, in a 600 bed-hospital, with a high volume of hematology and oncology patients. The ICU is a closed management but working in coordination with the hematology department. Every day, hematologist and intensivist met to manage ICU hematological patients. Criteria for ICU admission remained stable throughout the study, based on clinical needs goal of care decided with patient, intensivist and hematologist. According to the French law, consent was waived for such study. The study was approved by the ethic committer of SRLF (French Intensive Care Society) (CE-SRLF 16–54).

Patient characteristics were collected from medical records, including demographics, performance status before ICU admission, previous comorbidities (cardiac, respiratory, kidney diabetes mellitus), MM characteristics (date of diagnosis, number of lines of treatment before ICU admission, severity at diagnosis) and previous treatments (name of chemotherapy, autologous stem cell treatment, new molecules treatment). Newly diagnosed MM was defined as MM diagnosed within one month before ICU admission or during ICU stay. Data concerning ICU stay included SAPSII and SOFA scores, reasons for admission, delay between hospital and ICU admission, life-support treatments (mechanical ventilation, vasopressors, renal replacement therapy), infections at ICU admission or during ICU stay and outcomes (ICU, hospital, and one-year mortality). Follow-up was performed from the hospital database.

Staging for MM was assessed with cytogenetic abdnormalities in the more recent period [14]: high risk was corresponding to plasma cell leukemia or MM with del (17p), TP53 mutation, t (4; 14), t (14; 16) or t (14; 20) co-occuring with +1q or del (1p32), monoallelic del (1p32) with +1q or biallelic del (1p32), high β2 microglobulin when available with normal creatinine, Standard risk corresponded to other cytogenetics abdnormalities. Because cytogenetics characteristics were not available in the first period, the Salmon–Durie classification was used to stratify patients [15]. High risk MMs were defined by Salmon–Durie classification stage III.

### Statistical analysis

The primary objective was to compare one-year mortality between patients admitted during the first period (2007–2015) and during the second period (2016–2023).

The secondary objectives were to identify clinical and disease-related factors associated with mortality during the most recent period.

In this retrospective study, number of included patients was not defined. However, we decided to include patients within 8 years around 2015 considering that most of major changes in MM occurred during this period. Only the first ICU admission was considered.

All data were expressed as n (%) or median (IQR) as required.

Patients admitted during the first and second periods were compared with univariate analysis. Variables at ICU admission associated with one-year mortality or variables including high differences between the 2 periods, in univariate analysis were included in multivariate analysis unless there was more than 20% non available data (Performans status, kidney disease before admission). Some variables were correlated and only one of them was included in the analysis (time from MM diagnosis to ICU and number of treatment lines, SOFA and SAPS2 scores). Because of the relation between type of treatment and period, none of MM treatment were included in the survival analysis. Because sepsis during MM remains one of the most important reason of ICU admission with impact on mortality [16], the reason of ICU admission was forced in the Cox model. Although inaugural MM status was associated with mortality in univariate analyses, it was not included in the multivariate analysis because this variable, was already included in number of line before ICU admission. Data included in the Cox model analysing factors associated with mortality were age, duration of MM (more than 2 lines treatment before ICU admission), diabetes, cardiovascular disease, pulmonary disease, kidney amylosis, duration between hospital and ICU admission, severity of acute disease (SOFA score at ICU admission) and reason of ICU admission (Shock vs acute respiratory failure avs other reason). Cut-off for continuous variables were median value found in the total population. Cox model was performed for mortality analysis.

First non-adjusted survival curves were performed for the two periods. Then adjusted curves with age, comorbidities, lines of treatment before ICU admission, time from hospital and ICU admission,SOFA score at ICU admission and reason for ICU admission (shock vs acute respiratory failure vs other reason) were performed according to the period of admission.

Secondary objectives were to described factors associated with one-year mortality in the most recent period (2016−2023). During this period, patients with no data concerning D365 mortality were not included in the analyses. Variable at ICU admission associated with D365 mortality in univariate analysis were included in multivariate analysis unless there was more than 20% non available data (no variable). Some variables were correlate and only one of them was included in the analysis (time from MM to ICU and number of treatment lines, SOFA and SAPS2 score).Because of the relation between type of treatment, the year of ICU admission and the number of treatment lines, none of MM treatment were included in the analysis. Data included in the multivariate analysis for factors associated with mortality were age, diabete, duration of MM (i.e more than 2 lines treatment before ICU admission), severity of acute disease (SOFA score at ICU admission) and reason of ICU admission. Cox model was performed for mortality analysis.

All analyses were carried out with software R, version 3.6.2. The ‘Table One’, ‘Survival’, Survminer’ and ‘adjusted Curve’, ‘ggplot2’ packages were used to perform the analyses. No imputation for missing data was performed.

## Results

From 2007 to 2023, 428 patients were included: 199 patients within the 2007–2015 period and 229 patients within the 2016–2023 period (Figure 1S in supplementary data). Median age was 64.9 (57.8−71.2) years-old, gender was female for 173 (40.4%) patients. Most of patients had at least one comorbidity, mainly cardiac (n = 220, 51.4%) and diabete (n = 66, 15.4%).

Table 1 describes patients’ characteristics at ICU admission. Patients had received 1(1-3) lines of treatment for MM before ICU admission, including 172 (40.2%) patients receiving autologous stem cell transplant. Myeloma was diagnosed the month before ICU admission in 82 (19.2%) patients.

**Table 1:** Characteristics at ICU admission according to the admission period.

	Total	2007−2015	2016−2023	
Variables	(n = 428)	(n = 199)	(n = 229)	**p**
Baseline characteristics				
Age (year) m, (IQR]	64.9 (57.8−71.2)	63.9 (57.8−71.0)	65.5 (57.8−71.4)	0.52
Gender female	173 (40.4)	81 (40.7)	92 (40.2)	0.96
Performans status (NA = 205)	1 (0−1)	1 (1−1)	1 (0−1)	0.008
**Comorbidity**				
Cardiac	220 (51.4)	91 (45.7)	129 (56.1)	0.04
Pulmonary	61 (14.3)	16 (8.0)	43 (18.8)	0.001
Kidney (NA = 182)	52 (21.1)	7 (3.5)	45 (19.7)	<0.001
Diabete mellitus	66 (15.4)	23 (11.6)	43 (18.7)	0.06
SAPSII score	43 (34−55)	43 (37−55)	43 (32−54)	0.33
SOFA score (median (IQR))	5 (2−7)	4 (2−7)	5 (3−8)	0.002
Characteristics of myeloma				
Delay from diagnosis (months) Severity	25.9 (2.2−70.2)	41.7 (13.4−80.4)	10.2 (0.93−59.5)	<0.001
High risk multiple myeloma			119 (52.0)	
Salmon-Durie III		142 (71.4)		
**Disease progression**				
Newly diagnosed myeloma	82 (19.2)	23 (11.6)	59 (25.7)	0.001
Number of treatment lines before ICU admission	1 (1−3)	2 (1−2)	1 (1−3)	0.52
Heart amyloidosis	15 (3.7)	6 (3) (NA = 3)	9 (3.9)	0.16
Kidney amyloidosis	13 (3.1)	6 (3) (NA = 3)	7 (3)	0.17
Autologous stem cell transplant	172 (40.2)	81 (40.7)	91 (39.7)	0.96
Allogenic stem cell transplant	15 (3.5)	12 (6) (NA = 3)	3 (1.3)	0.01
**Reason of ICU admission**				0.001
Shock	97 (22.7)	25 (13.7)	68 (29.7)	
Acute respiratory failure	170 (39.7)	85 (46.7)	81 (35.2)	
Other	161 (37.6)	81 (40.7)	80 (34.9)	
Delay from hospital to ICU admission	2 (1−9)	1 (0−7)	2 (0−11)	0.06
**Life-sustaining treatments in ICU**				
NIV	67 (15.7)	31 (15)	36 (15.7)	1
Invasive mechanical ventilation	115 (26.9)	55 (27.6)	60 (26.1)	0.82
Vasopressors	105 (24.5)	44 (22.1)	61 (26.6)	0.33
RRT	97 (22.7)	53 (26.6)	44 (19.2)	0.09
ICU length of stay	3 (2−6)	3 (2−6)	4 (2−7)	0.32
End of life decision	41 (9.6)	8 (4)	33 (14.4)	<0.001
**Infections at ICU admission or during ICU stay**				
Pneumonia	156 (36.4)	65 (32.7)	91 (39.7)	0.15
Other infection	80 (18.8)	39 (19.6)	41 (17.9)	0.25
Catheter-related sepsis	22 (5.1)	10 (5)	12 (5.2)	1
**Outcome**				
ICU mortality	50 (12.1)	25 (12.6)	25 (10.9)	0.69
Hospital mortality	93 (21.7)	46 (25.3)	47 (20.4)	0.35
D365 mortality	170 (40.6)	88 (44.3) (NA = 5)	82 (35.8) (NA = 4)	0.15

SAPSII score: simplified acute Physiology score; SOFA score: Sequential Organ Failure Assessment, NIV: non-invasive ventilation, RRT: Renal replacement therapy.

*Other: Coma (n = 33), Kidney failure (n = 75), hyperviscosity (n = 23), hemorraghe (n = 6), other miscellaneous reason (n = 24).

The main reasons for ICU admission were acute respiratory failure (n = 170, 39.7%). During ICU stay, almost one quarter of patients received renal replacement therapy (n = 97, 22.7%) and 115 (26.9%) needed invasive mechanical ventilation. At ICU admission or during ICU stay, 156 (36.4%) patients had pneumonia, 80 (18.8%) patients had other infection (mostly digestive or urinary infections). (Table 1S).

Mortality at ICU discharge, hospital discharge and one year were respectively 12.1% (n = 50), 21.7% (n = 93), 40.6 % (n = 170). Follow-up duration after ICU discharge was 10.45 (1.26–33.3) months. Table 2S describes patients according to the one-year outcome.

**Fig. 1:**
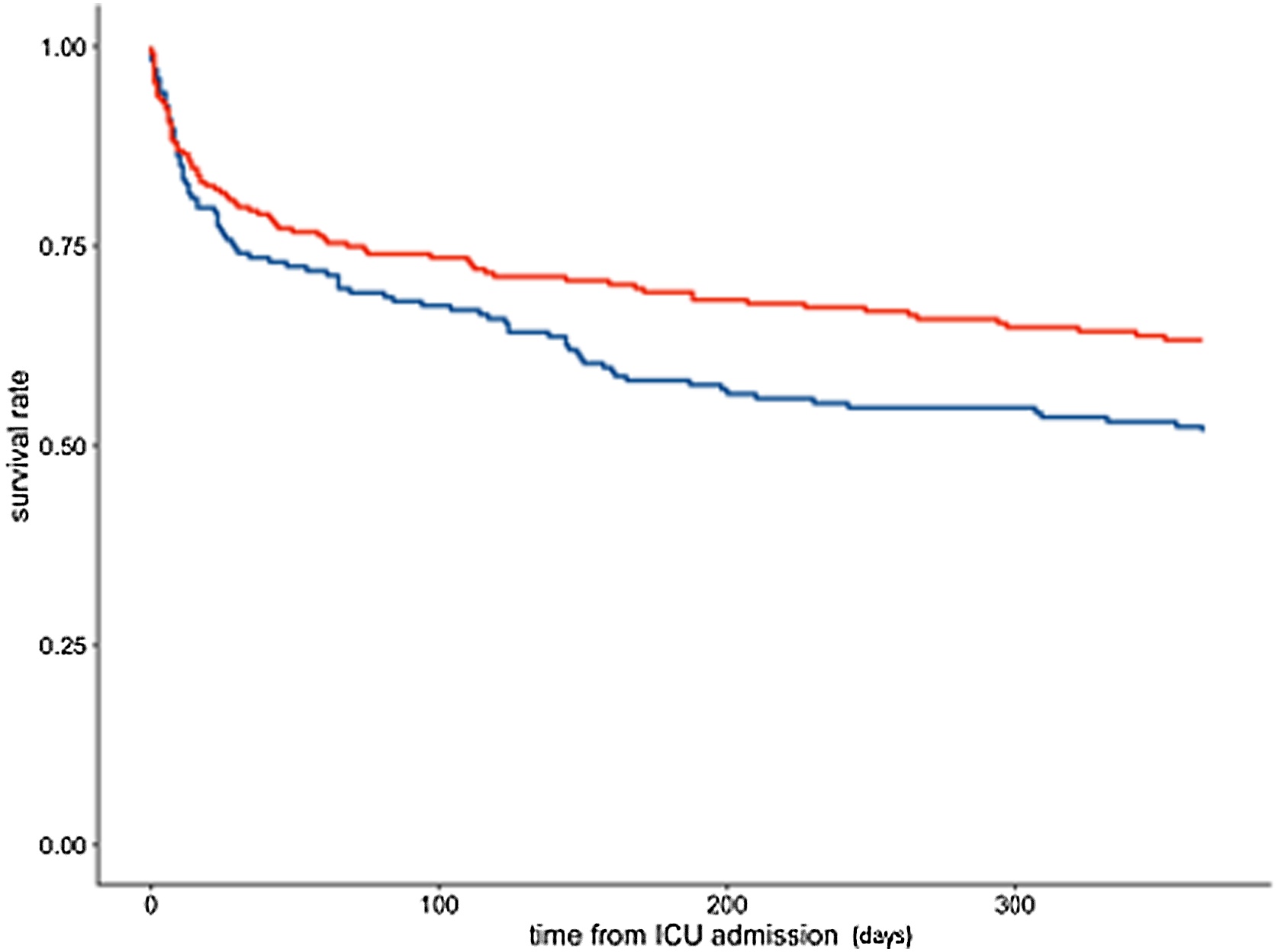
Survival curves adjusted with age, comorbidities, lines of treatment before ICU admission, time from hospital and ICU admission,SOFA score at ICU admission and reason for ICU admission. Blue curve: 2007−2015 cohort. Red curve: 2016−2023 cohort.

Comparing to first period, patients from the recent period (2016−2023) were more severe at ICU admission: SOFA score were respectively in the recent and first period of 5 (3−8) vs 4 (2−7), p < 0.001. Patients had shorter treatment duration before ICU admission (10.2 (0.93−59.5) vs 41.7 (13.4−80.4) months, p < 0.001). Reason for ICU admission were significantly different between the two periods (p < 0.001). Acute respiratory failure was the most frequent reason for ICU admission in the two periods but shock as reason for ICU admission increased un the second period (Table 1). Crude one-year mortality was not different between the two periods (HR = 0.76 (0.56–1.03), p = 0.07). After adjustement one year mortality was lower in the recent period compared to first period (HR = 0.68 (0.49−0.95), p = 0.02) (Table 2, Fig. 1)

**Table 2:** Factors associated with one year mortality.

Characteristic at ICU admission	HR (IC95%)	P
2016−2023 ICU admission	0.68 (0.49−0.95)	0.02
Age	1.02 (1.00−1.04)	0.003
Diabetes	1.02 (0.66−1.58)	0.9
Heart disease	0.79 (0.57−1.09)	0.16
Pulmonary disease	0.76 (0.51−1.33)	0.27
More than 2 treatment lines before ICU admission	2.20 (1.59−3.04)	<0.001
Kidney amyloidosis	2.03 (0.97−4.22)	0.05
Time from hospital admission to ICU admission >1 day	1.02 (1.01−1.03)	<0.001
SOFA score	1.13 (1.08−1.18)	<0.001
Reason for ICU admission		
Shock	0.91 (0.58−1.42)	0.67
Acute respiratory failure	1.50 (1.04−2.16)	0.02
Other*	1	

SOFA score: Sequential Organ Failure Assessment.

* Other: Coma (n = 28), Kidney failure (n = 67), hyperviscosity (n = 22), hemorraghe (n = 6), other miscellaneous reason (n = 23). Unknown status at one year (n = 9).

### Patients from the recent cohort

Among the 229 patients included in the recent cohort (2016−2023), 225 (98.2%) patients were not lost-of follow-up at one-year, Table 3 summarized patients’ characteristics at ICU admission according to the survival status at one year. Within this period, most of patients received proteasome inhibitors. Small number of patients had received bispecific antibody before ICU admission (4 patients).

**Table 3:** Characteristics of ICU admitted patients according one year mortality in the recent period.

	Dead at D365	D365 survivors	
Variables	(n = 82)	(n = 143)	p
Baseline characteristics			
Age (year) m, (IQR]	67.4 (61.9−75.1)	64.9 (56,5−69.3)	0.006
Gender female	34 (41.5)	57 (39.9)	0.92
Performans status	1 (0−2)	1 (0−1)	<0.001
Comorbidity			
Cardiac	45 (54.9)	83 (58)	1
Pulmonary	16 (19.5)	29 (20.3)	0.41
Kidney	25 (30.5)	20 (14)	0.005
Diabete mellitus	22 (26.8)	21 (14.7)	0.04
SAPSII score	52 (38−66)	39 (31−50)	<0.001
SOFA score (median (IQR))	7 (4−9)	4 (2−7)	<0.001
Characteristics of myeloma			
Delay from diagnosis (months)	25 (2−79.9)	6.9 (0.42−45.2)	0.005
High risk multiple myeloma	39 (47.6)	76 (53.1)	0.31
Disease progression			
Newly diagnosed myeloma	16 (19.5)	40 (28)	0.26
Number of treatment lines before ICU admission	2(1−4)	1 (1−2)	<0.001
Heart amylosis	4 (4.9)	5 (3.5)	0.87
Kidney amylosis	4 (4.9)	3 (2.1)	0.44
Autologous stem cell transplant	31 (37.8)	59 (41.3)	0.71
Allogenic stem cell transplant (NA = 3)	1 (1.2)	2 (1.4)	1
Treatment for myeloma			
Steroid	72 (87.8)	130 (90.9)	0.6
Bortezomib	65 (79.3)	108 (75.5)	0.63
Cyclophosphamide	46 (56.1)	51 (35.7)	0.005
Lenalidomid	44 (53.7)	60 (42)	0.12
Pomalidomide	23 (28)	21 (14.7)	0.02
Carlfilzomib	15 (18.3)	14 (9.8)	0.1
Ixazomib	1 (1.2)	5 (3.5)	0.55
Teclistamab	1 (1.2)	1 (0.6)	1
Erlnatamab	1 (1.2)	0	NA
Isatuximab	1 (1.2)	1 (1.2)	1
Masitinib	0	1 (0.4)	NA
Reason of ICU admission			0.31
Shock	25 (30.5)	43 (30.1)	
Acute respiratory failure	34 (41.5)	47 (32.9)	
Other	23 (28)	53 (37.1)	
Delay from hospital to ICU admission	2.5 (0−12)	1 (0−9)	0.19
Life-sustaining treatments in ICU			
NIV	18 (22)	18 (12.6)	0.09
Invasive mechanical ventilation	35 (42.7)	25 (17.5)	<0.001
Vasopressors	32 (39)	29 (20.3)	0.004
EER	25 (30.5)	18 (12.6)	0.002
ICU length of stay	3.5 (2−8.7)	4 (2−6)	0.68
End of life decision	29 (35.4)	4 (2.6)	<0.001
Outcome			<0.001
ICU mortality	25 (30.5)	–	NA

SAPSII score: simplified acute Physiology score; SOFA score: Sequential Organ Failure Assessment, NIV: non-invasive ventilation, RRT: Renal replacement therapy.

Unknown status at one year (n = 4).

*Other: Coma (n = 9), Kidney failure (n = 34), hyperviscosity (n = 23), hemorraghe (n = 5), other miscellaneous reason (n = 11).

Multivariate analysis identified two independent risk factors of mortality: more than two lines of MM treatment before ICU admission (HR 1.77; 95% CI: 1.06–2.95; *p* = 0.02), SOFA score (HR 1.14; 95% CI: 1.06–1.22; *p* < 0.001). Performans status over 2, age and diabete were no longer associated with survival (Table 4).

**Table 4:** Factors associated with one year mortality during the period 2016-2023.

Characteristic at ICU admission	HR (IC95%)	P
Age	1.02 (0.99−1.05)	0.13
Performans status >2	1.40 (0.61−3.23)	0.42
More than 2 treatment lines before ICU admission	1.77 (1.06−2.95)	0.02
Diabete	1.46 (0.84−2.56)	0.17
SOFA	1.14 (1.06−1.22)	<0.001
Reason for ICU admission		
Shock	0.71 (0.37−1.36)	0.31
Acute respiratory failure	1.14 (0.63−2.06)	0.66
Other	1	

*Other: Coma (n = 5), Kidney failure (n = 32), hyperviscosity (n = 22), hemorraghe (n = 4), other miscellaneous reason (n = 10).

Unknown status at one year (n = 4).

SOFA score: Sequential Organ Failure Assessment.

## Discussion

In this large retrospective monocentric cohort of critically ill, MM patients admitted to the ICU between 2007 and 2023, we observed significant shifts in both patient profiles and outcomes. Also, those data confirmed that survival of patients with MM improved for those who need ICU admission. Indeed, mortality rate still decreased in the cohort compared to old cohorts from years before 2005’ [11]. Moreover, compared to earlier years, patients admitted during the recent period (2016–2023) had shorter time from diagnosis to ICU admission, were more likely to be newly diagnosed, and had received fewer prior lines of treatment. Despite higher organ failure scores (SOFA), these patients demonstrated slightly lower mortality rate, reflecting possible improvements in ICU care and hematological disease management during and after ICU stay.

Our findings are consistent with earlier reports showing that hematological patients increasingly benefit from critical care interventions [12,13]. However, we also identified persistent risk factors of poor outcomes. In multivariable analysis, high level of hematological treatment and more severe organ failures at ICU admission remained independently associated with one-year mortality. These findings support the need for early ICU admission in these patients and close collaboration with hematologists to identify those most likely to benefit from intensive care management [[17], [18], [19]]. Indeed, Figs. 2 confirmed that survival has improved over time, even after adjustments for age, comorbidities, severity of acute disease and prior treatment burden, delayed admission, amyloidosis and reason for ICU admission. These improvements likely reflect the combined effects of earlier ICU referral, optimized organ support, and advances in multiple myeloma therapy, including the introduction of next-generation agents such as carfilzomib, pomalidomide, and monoclonal antibodies. These results are consistent with a recent study performed on MIMICIII database. Although the authors did not analyze long-term survival and performance status before ICU admission, this study reporting 126 patients with MM admitted to ICU found that severity of critical illness and disease status was associated with hospital mortality [17]. Also, recent French abstract describing 72 patients in a cohort study, confirmed that severity of critical illness and number of previous treatment lines were associated with D90 mortality [20].

Interestingly, in the recent period high-risk cytogenetics was not associated with excess mortality, suggesting that cytogenetic risk may not be relevant for short-term ICU survival and could not be decisive for ICU admission in these patients. This observation supports more inclusive ICU admission policies for patients with biologically aggressive disease. [12].

The reasons for ICU admission remained dominated by respiratory failure and shock, in line with previous literature [11,17,20]. However, life-sustaining interventions such as mechanical ventilation and vasopressor use were more selectively applied in recent years, perhaps reflecting improved triage and early intervention strategies. End-of-life decisions were markedly more common among non-survivors, reinforcing the need for early goals-of-care discussions in patients with limited reversibility [19,21].

This study has several strengths, including a large sample size, real-world data, and extended follow-up. However, this study had several limitations. First, this was a retrospective design and single-center cohort. Selection bias and unmeasured confounders, such as frailty or socioeconomic status, may have influenced outcomes. Second, only one-year mortality was assessed and long-term functional outcomes or quality of life were not recorded [22,23]. Third, only patients referred and admitted to ICU were analyzed. Patients who were judged to sick or too well to be admitted to ICU were not analyzed [18]. Also, the increased survival rate in the recent period may reflect a better triage before ICU admission or earlier admission [24]. Moreover, there was no data concerning the time between symptoms onset and ICU admission. Indeed, longer length of symptoms has been associated with higher mortality rate for hematological patients [25]. These factors may have modified some results. Lastly, severity of infections during ICU stay was not detailed. Particularly septic shock usually associated with mortality in the setting of MM, was not described. However, all organ failures occurring during ICU stay were recorded. Multivariate analysis including pneumonia did not modify the results concerning one-year mortality (data not shown).

In conclusion, our study supports the evolving role of intensive care in the management of patients with multiple myeloma. While survival remains guarded in those with advanced disease or severe critical illness, overall outcomes have improved in recent years. Early ICU referral and individualized support strategies, coupled with ongoing advances in myeloma treatment, appear to be key drivers of these improved outcomes. Future studies should focus on integrating ICU care into the broader continuum of multiple myeloma.

## Authors' contributions

SN, VL, AK designed the study, included patients and write de the manuscript, VL and MD performed the statistical analysis, SH, LC, EA, HK discussed the results and revised the manuscript.

## Consent for publication

NA.

## Ethics approval and consent to participate

According to the French law, consent was waived for such study. The study was approved by the ethic committee of SRLF (French society of medical ICU) (CE-SRLF 16–54) in the method part.

## Funding

No funding source.

## Availability of data and materials

The datasets used and/or analyzed during the current study are available from the corresponding author on reasonable request.

## Declaration of competing interest

The authors declare that they have no known competing financial interests or personal relationships that could have appeared to influence the work reported in this paper.

## Footnotes

Appendix A Supplementary material related to this article can be found, in the online version, at doi:https://doi.org/10.1016/j.aicoj.2026.100100.
