# Supplementary material for: Improved survival in patients admitted to ICU with multiple myeloma: a retrospective cohort analysis
Source: Ann Intensive Care. 2026 Jun 13;16:100100. doi: 10.1016/j.aicoj.2026.100100 (PMC13330700; doi:10.1016/j.aicoj.2026.100100)
Supplement: Supplementary file 1 [file mmc1.pdf]

Supplementary material

Improved survival in patients admitted to ICU with multiple myeloma: a retrospective cohort analysis

Sabrine Nakaa[1], Leo Caillot, Akli Chermak[1], Helene Kemp, Stéphanie Harel, Michael Darmon[1], Elie Azoulay[1], Virginie Lemiale[1]

[1] Medical ICU, Saint Louis Hospital, 1 avenue Claude Vellefaux, 75010 PARIS

[2] Immuno-hematology, Saint Louis Hospital, 1 avenue Claude Vellefaux, 75010 PARIS

Table 1S : Infections at ICU admission or during ICU stay.

Table 2S : Characteristics at ICU admission according to the one-year outcome (NA=6).

SAPSII score: simplified acute Physiology score; SOFA score: Sequential Organ Failure Assessment, NIV: non-invasive ventilation, RRT: Renal replacement therapy

*Other : Coma (n=32), Kidney failure (n=72), hyperviscosity (n=22), hemorraghe (n=6), other miscellaneous reason (n=24)

Unknown status at one year (n=6)

Figure 1S Flowchart.

Table1S

Infection	microbiology	n
Pneumonia (n=156)	Encapsulated bacteria	8
	Gram negative bacteria	28
	Viral	44
	Fungal infection	7
	Tuberculosis	1
	Non documented	66
Catheter related infection		22
Other infection (n=51)	*S. Pneumoniae* meningitis	1
	N. Meningitis meningitis	1
	Gram positive cocci septicemia	8
	Urinary or abdominal gram negative bacteria	38
	Pseudomonas aeruginosa	5
	Non documented sepsis	5

Table 2S

Variables	Dead at D365 (n=170)	D365 survivors (n= 249)	p
**Baseline characteristics**			
Age (year) m, (IQR]	66.2 (59,1-72.5)	63.8 (56.8-70.0)	0.01
Gender female	65 (38.2)	107 (43)	0.38
Performans status (NA=205)	1 (0.2-2)	1 (0-1)	<0.001
Comorbidity			
Cardiac	84 (49.4)	131 (52.8)	0.78
Pulmonary	21 (12.4)	39 (15.7)	0.41
Kidney (NA=177)	26 (15.3)	26 (10.4)	0.009
Diabete mellitus	28 (16.5)	35 (14.1)	0.58
ICU admission during 2007-2015 period	88 (51.8)	106 (42.6)	0.079
SAPSII score	47.5 (38-63)	40 (33-51)	<0.001
SOFA score (median (IQR))	6 (3-9)	4 (2-6)	<0.001
Characteristics of myeloma			
Delay from diagnosis (months)	33.9 (6.3-76.9)	19.3 (1.65-62.5)	0.022
Disease progression			
Newly diagnosed myeloma	25 (14.7)	54 (21.7)	0.14
Number of treatment lines before ICU admission	2(1-3)	1 (1-2)	<0.001
Heart amylosis (NA=3)	6 (3.5)	9 (3.6)	0.11
Kidney amylosis (NA=3)	8 (4.7)	5 (2)	0.03
Autologous stem cell transplant	73 (42.9)	97 (39)	0.47
Allogenic stem cell transplant (NA=3)	9 (5.3)	6 (2.4)	0.25
Treatment for myeloma			
Steroid	141(82.9)	193 (77.5)	0.28
Bortezomib	76 (44.7)	118 (47.4)	0.79
Cyclophosphamide	97 (57.1)	96 (38.6)	0.01

Lenalidomid	47 (27.6)	63 (25.3)	0.02
Pomalidomide	48 (28.2)	38 (15.3)	0.003
Carfilzomib	15 (8.8)	14 (5.6)	0.02
Ixazomib	1 (0.6)	5 (2)	0.07
Teclistamab	0	1 (0.6)	NA
Elranatamab	1 (1.6)	0	NA
Isatuximab	1 (0.6)	1 (0.4)	NA
Masitinib	0	1 (0.4)	NA
Reason of ICU admission			0.31
Shock	38 (22.4)	59 (23.7)	
Acute respiratory failure	75 (44.1)	92 (36.9)	
Other*	57 (33.5)	98 (39.8)	
Delay from hospital to ICU admission	2.5 (0-10)	1 (0-8.5)	0.06
Infections at ICU admission or during ICU stay			
Pneumonia	70 (42.2)	84 (33.7)	0.15
Other infection (NA=2)	33 (19.4)	47 (18.9)	0.95
Catheter-related infection	13 (7.6)	9 (3.6)	0.11
**Life-sustaining treatments in ICU**			
NIV	34 (20)	32 (12.9)	0.06
Invasive mechanical ventilation	72 (42.4)	43 (17.3)	<0.001
Vasopressors	62 (36.5)	43 (17.3)	0.004
RRT	46 (27.1)	50 (20.1)	0.12
ICU length of stay	2.5 (0-10)	1 (0-8.5)	0.6
End of life decision	35 (20.6)	6 (2.4)	<0.001
**Outcome**			<0.001
ICU mortality	50 (29.4)	-	NA

Figure 1S

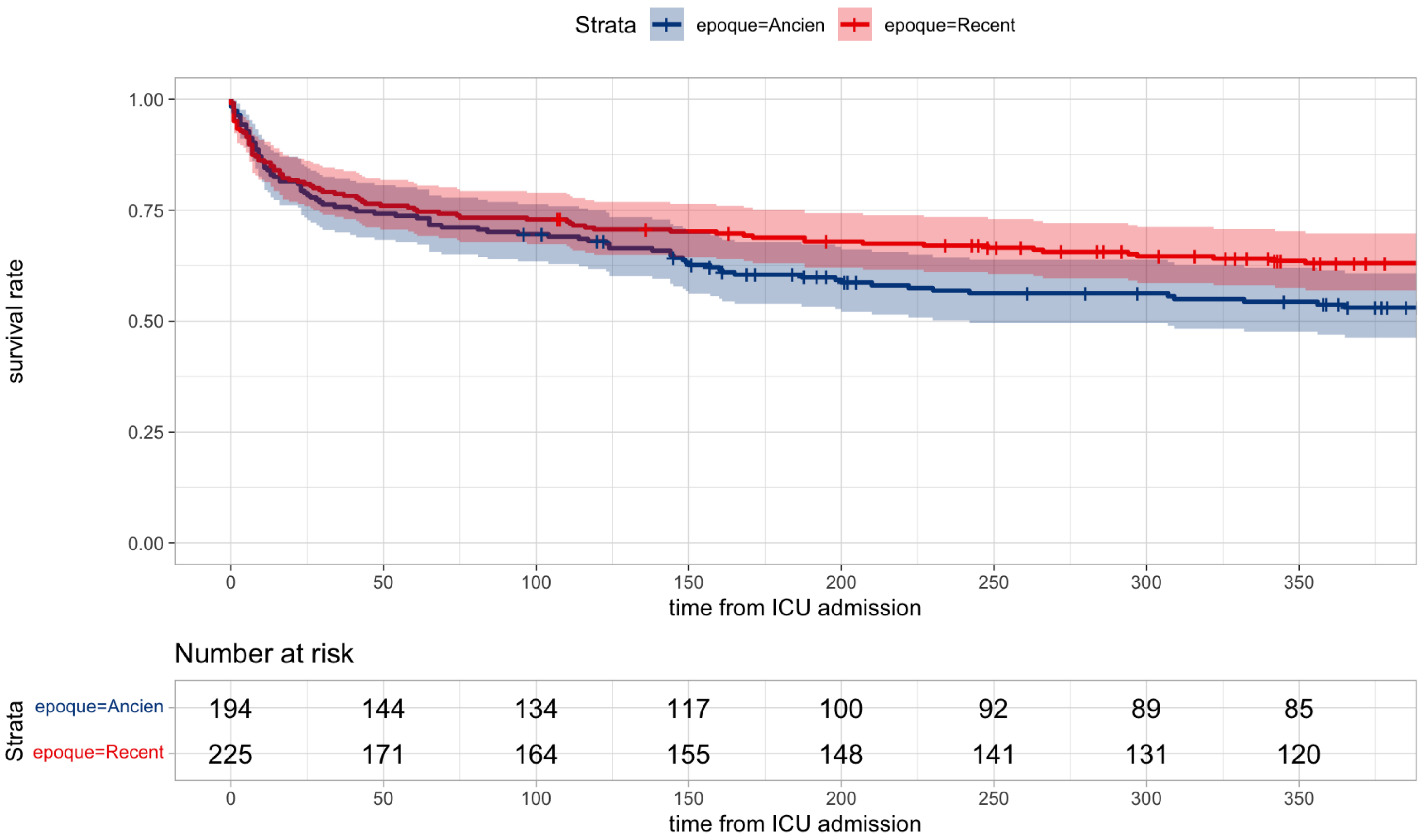

Strata
epoque=Ancien
epoque=Recent
survival rate
1.00
0.75
0.50
0.25
0.00
0
50
100
150
200
250
300
350
time from ICU admission
Number at risk
Strata
epoque=Ancien
epoque=Recent
194 144 134 117 100 92 89 85
225 171 164 155 148 141 131 120
0
50
100
150
200
250
300
350
time from ICU admission
